# COVID-19 and medical education in Africa: a cross sectional analysis of the impact on medical students

**DOI:** 10.1186/s12909-021-03038-3

**Published:** 2021-12-09

**Authors:** Alec Bernard, Gnendy Indig, Nicole Byl, Amani Nureddin Abdu, Dawit Tesfagiorgis Mengesha, Bereket Alemayehu Admasu, Elizabeth Holman

**Affiliations:** 1 University of Michigan Medical School, 48103 Ann Arbor, MI USA; 2 St.Paul’s Hospital Millennium Medical College, 1271 Swaziland Street, Addis Ababa, Ethiopia

**Keywords:** COVID-19, Pandemic, Africa, Health disparities, Medical education, Medical students

## Abstract

**Background:**

The African continent currently experiences 25% of the global burden of disease with only 1.3% of the world’s healthcare workers. The COVID-19 pandemic has caused unprecedented disruption to medical education systems, increasing the strain on already-vulnerable regions. Our study examines the impact of COVID-19 on medical students across 33 countries in the African continent.

**Methods:**

A 39-item anonymous electronic survey was developed and distributed to medical students across Africa through social networks to assess the impact of the COVID-19 pandemic on medical education. The survey assessed the domains of: class structure changes and timing, patient interactions, exam administration, learning environment satisfaction, mental health impacts, and volunteer opportunities/engagement.

**Results:**

694 students across 33 countries participated. 80% of respondents had their classes suspended for varied lengths of time during the pandemic, and from these students 59% of them resumed their classes. 83% of students felt they were in a supportive learning environment before the pandemic, which dropped to 32% since the start. The proportion of students taking exams online increased (6–26%, p<0.001) and there was a decrease in the proportion of students seeing patients as a part of their education (72–19%, p<0.001).

**Conclusions:**

COVID-19 is harming medical students in Africa and is likely to worsen the shortage of the future’s healthcare workforce in the region. Pandemic-related impacts have led to a degradation of the learning environment of medical students. Medical schools have shifted online to differing degrees and direct patient-care in training of students has decreased. This study highlights the urgent need for flexible and innovative approaches to medical education in Africa.

**Supplementary Information:**

The online version contains supplementary material available at 10.1186/s12909-021-03038-3.

## Background

The pandemic-related disruption to medical education has worrisome implications for global public health. In Africa, 1.3% of the world’s health care workers (HCW) care for people who experience 25% of the global burden of disease [1]. Africa has 2.3 HCW per 1000 population, compared with 25 per 1000 in the Americas. Estimates suggest that the current training is insufficient to even maintain the current density of HCW [2]. This shortage is likely to be exacerbated in parts of the world less able to adapt their medical education systems to the demands of the COVID-19 pandemic. At the time of writing, COVID-19 has infected over 203 million people with over 4.3 million deaths, causing unprecedented disruption to health and education systems [3]. Eleven countries in Africa lack even a single medical school, and 24 countries have only one medical school [4]. There are estimated to be a total of 143 medical schools in all of Sub-Saharan Africa graduating around 10,000 physicians annually [5]. To date, there is no comprehensive information about the impact of COVID-19 and related mitigation measures on medical education in Africa. Our study serves to fill this gap by examining the impact of the pandemic on medical students across the African continent.

According to the United Nations Educational, Scientific and Cultural Organization (UNESCO), at its peak, the pandemic affected over 75% of students across the world, with a higher percentage impacted in Africa [6]. The reactions of medical school administrators to this unprecedented stressor have varied, ranging from pausing all educational activities, to graduating students early to assist in fighting the pandemic [7, 8]. Additionally, E-learning has emerged as a central strategy in continuing education in the era of COVID-19 [9]. However, many low- and lower-middle income countries (LMIC) struggle with e-learning due to challenges with infrastructure, resource availability, communication, and social barriers [10]. A recent review of the transition to e-learning in pharmacy schools in Africa found numerous barriers related to poor accessibility in rural areas, high cost of internet data, and poor infrastructure in many areas [11, 12].

Furthermore, students are likely to face new challenges in the learning environment. Even before the pandemic, medical students were already at a greater risk for mental health problems than their matched peers [13]. The shift away from in-person education deprives students of social and professional interactions to the possible detriment of their education and mental well-being and we must understand the ways the pandemic has impacted students’ learning environment.

Disruptions in medical training have the potential for largely detrimental impacts on the public health of a region already facing a disproportionate burden of disease. A thorough understanding of the disruption caused by the COVID-19 pandemic is necessary for the development of policies to limit the harm of this and future stressors to ensure the continued health of a region already facing a devastating shortage of trained providers. We examined medical students’ perspectives of the impact of the pandemic on their medical education across various domains in order to inform the development of future policy. Specifically, we strove to answer the following questions: Was medical education suspended during the COVID-19 pandemic in Africa? How were the learning environment, student mental health, and perceptions of safety impacted? And was there a shift in the format of classes?

## Methods

Study Design: While there are long-standing rigorously validated survey instruments like the Dundee Ready Educational Environment Measure (DREEM) for use in health professions education, existing instruments may lack content validity since they were not developed with this unprecedented global stressor in mind [14]. A review of existing medical education survey instruments and COVID-19 surveys cataloged on the PhenX Toolkit website [15] determined that there was no single instrument that appeared well targeted to assessment of medical education during the COVID-19 pandemic in Africa. Accordingly, the decision was made to develop a novel survey instrument to conduct research specific to the impact of COVID-19 on medical education in Africa. Given the highly situational nature of this aim, we believe that a new instrument was needed in order to enhance key measurement properties like content and construct validity.

Experts in survey methodology and medical education at the University of Michigan Medical School (UMMS) developed a 39-item survey assessing the following domains: class structure changes and timing, patient interactions, exam administration, learning environment satisfaction, mental health impacts, and volunteer opportunities/engagement. The survey development team consisted of all authors, two professors of medical education, and faculty members of the UMMS office of evaluation and assessment. The survey was composed of Likert scale, dichotomous, and free-response items. It was modified with the assistance of students in St. Paul’s Millennium Medical College (SPHMMC) through pilot administration and cognitive interviewing to ensure readability and ease of understanding in multiple countries. The survey underwent three rounds of pilot administration and interviewing with four students at SPHMMC per round. The survey was edited for readability and ease of understanding between each round. Full survey available in Additional file 1.

 IRB approval was granted via St. Paul’s Millennium Medical College Institutional Review Board. All research was performed in accordance with the Declaration of Helsinki and approved by an appropriate ethics committee. Informed consent to participate was elicited prior to survey administration with an option to opt-out prior to survey administration.

Survey Distribution: This anonymous electronic survey was sent to medical students across African countries to assess the impact of the COVID-19 pandemic on medical students (Google Forms, CA). Survey administration took place from September 15th to October 5th, 2020. The survey was widely distributed throughout Africa via the International Federation of Medical Students Association (IFMSA) social network groups, including Facebook, WhatsApp, and Telegram. Participants were medical students, ranging from pre-clinical to final year medical students. The IFMSA is one of the largest medical student organizations with 1.3 million students across 123 countries worldwide, including all but 14 countries in Africa. Student representatives from each country apply for membership in IFMSA and after a 1-year probationary period, become full members. Representatives of IFMSA were asked to distribute the survey further within their medical schools to reach respondents who were not directly members of IFMSA.

### Data analysis

Quantitative analysis was completed using IBM SPSS version 27. Descriptive statistics were calculated for sociodemographic variables, as well as responses to each survey question. A Wilcoxon Signed Rank test was used to assess the differences between pretest and posttest responses. A Mann-Whitney U test was used to determine the pandemics impact on students’ mental health. Responses were analysed in aggregate.

## Results

**Fig. 1:**
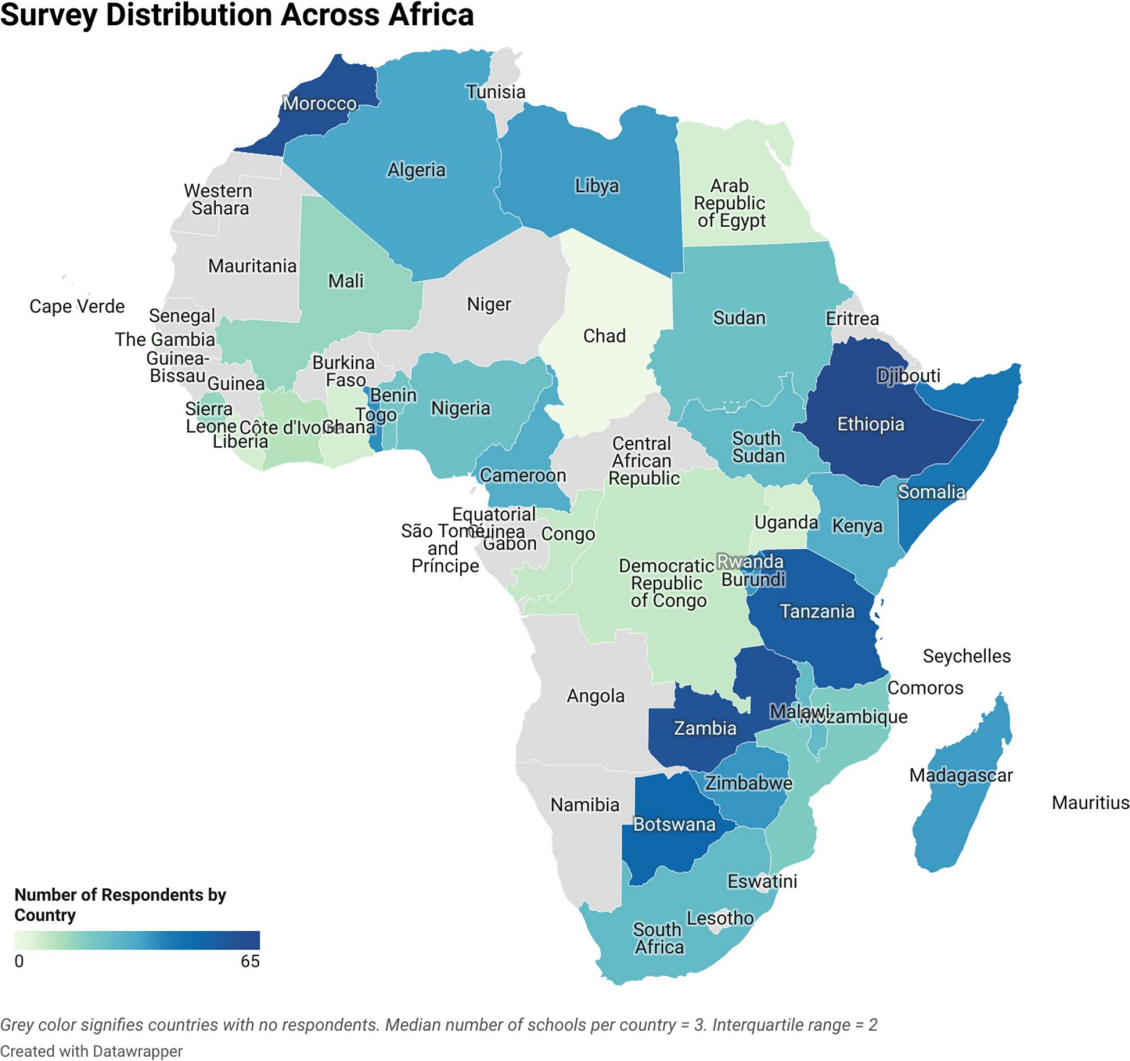
Locations of medical students surveyed across the African continent

Sociodemographic information of survey respondents is shown in Table 1. The locations of medical students represented in our sample are shown in Fig. 1.

Respondents represent 107 medical schools across 33 countries. The median medical schools per country is 3 with interquartile range of 2. The median respondents per medical school is 5 with interquartile range of 6.5. There are a total of 143 medical schools in Africa, with around 10,000 graduating physicians annually [5]. The nature of our social media recruitment does not allow estimates of the total number of students contacted by our study.

**Table 1:** Sociodemographic information for the study sample

Variable	*Frequency* *(n=694)*
Sex	
Male	335 (48%)
Female	347 (49%)
Prefer not to say	11 (2%)
No Response	1 (0%)
Current Enrolment Phase*	
Preclinical Education	194 (28%)
Clinical Education	490 (71%)
No Response	10 (1%)
Age in Years	
*Median*	23
*Interquartile Range*	3

*Generally, the first two years are preclinical education, followed by at least partial clinical education in years three and beyond

### Educational disruption due to COVID-19

**Fig. 2:**
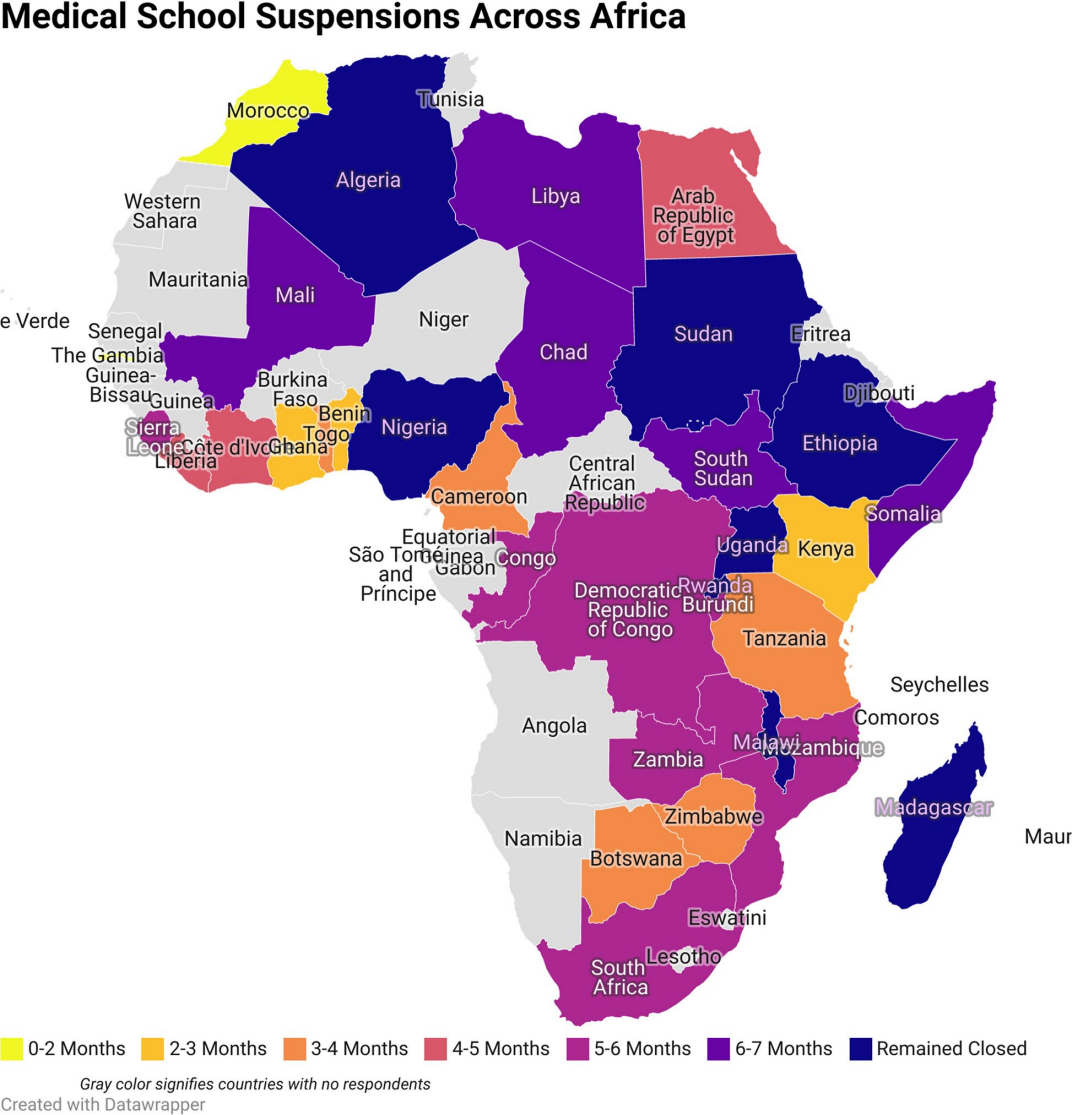
Average Length of Medical School Closures Across the African Continent

When asked about disruptions to their education, the majority of students, 80%, had their classes suspended during the pandemic, and from these students 59% of them resumed their class by the time of the study. Of the students whose classes were suspended and resumed, 70% of students reported online classes, with 19% reporting hybrid classes. 17% of students resumed their education in person. Among students without suspended classes, 50% reported a change to online coursework and 26% continued in person. There were no significant differences in the proportion of pre-clinical and clinical students who had their classes suspended (p=0.067), who resumed classes (p=0.061), or who endorsed a change in format after resumption (p=0.870). Medical school closures also varied significantly in length and the average length of closure by country is presented in Fig. 2.

### Perspective of learning environment before and after COVID-19 related disruptions

Students were asked about the learning environment. Overall, before the pandemic 83% of students felt they were in a supportive learning environment and since the pandemic, only 32% felt they were in a supportive learning environment. Even of the students who felt they were in a supportive learning enviroment before the pandemic, 67% felt that they were not in a supportive environment since the pandemic began.

**Fig. 3:**
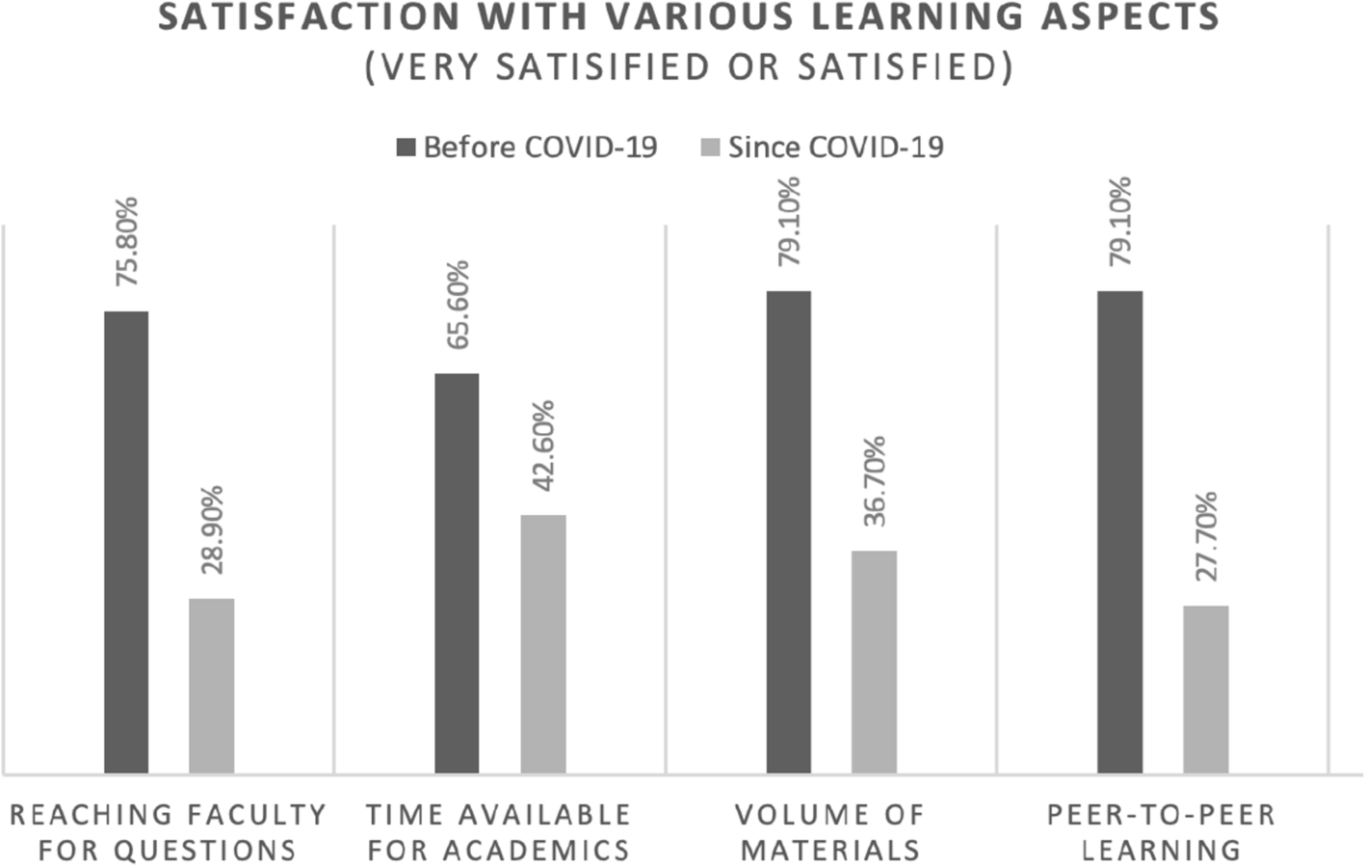
Proportion of sample reporting high satisfaction with various aspects of the learning environment and proportion of exams administered online before and after the start COVID-19 pandemic

Specifically, students were asked about their satisfaction levels with varying aspects of the teaching environement before and during the COVID-19 pandemic. After the start of the pandemic, 33% of students reported being “very dissatisfied” or “dissatisfied with the ease of reaching faculty for questions compared with 8% of students reporting dissatisfaction before the pandemic (p<0.001). Before COVID-19, 6% of students reported dissatisfaction with faculty engagement. In contrast, since the start of the pandemic 37% of students were dissatisfied with faculty preparedness for classes. The change in satisfaction levels with aspects of the learning environment and the changes in online exams are shown in Fig. 3. When asked about positive impacts of the changes in the learning environment, 57% of students selected increased flexibility of time and 38% selected decreased time spent community. Conversely, when discussing the negatives of the pandemic related changes, students cited technological issues (65%), decreased connection with their classmates (60%), and decreased availability of professors (50%).

When asked about other aspects of the learning envirnoment, 43% were satisfied with time available for academics, compared with 66% before the pandemic began (p<0.001). 37% of students were satisfied with the volume of material, compared with 79% before the pandemic (p<0.001). Students were also less satisfied with peer-to-peer learning opportunities, with 28% reporting satisfaction compared with 79% before the pandemic (p<0.001).

### Curricular structure changes related to COVID-19 Pandemic

Students reported several changes to the structure of their education. The proportion of students taking exams online increased from 6 to 26% since the start of the pandemic (p<0.001). There was additionally a decrease in the proportion of students seeing patients as a part of their education, from 72 to 19% (p<0.001). Among students who were seeing patients before the pandemic, 74% were no longer seeing patients since the start of the pandemic.

### Volunteer activities during the COVID-19 pandemic

Additionally, 29% of students reported engaging in volunteer activities during the pandemic. These activities included health screening work at hospitals and dormitories, contact tracing, fundraising for charitable causes, educational campains and volunteer tutoring.

### Effect of pandemic related changes to mental health

Students reported differering impacts of the pandemic on their mental health, with 44% reporting a negative change in their mental health, 23% reporting a positive change, and 34% reporting no change. Class suspensions were not significantly assoicated with impact on mental health (p=0.332). There were no significant associations between mental health and sex of respondent (p=0.519).

## Discussion

COVID-19 and associated mitigation measures are harming medical students in Africa, a region already suffering from a severe shortage of trained healthcare workers. The disruption to medical education has similarities and differences from the impact in high-income countries (HIC) countries such as the United States (US). In the US, medical schools are able to follow guidance from the Association of American Medical schools. This guidance included a temporary pause to in-person clinical rotations and shift towards online education [16]. Standards and timelines for testing and graduation requirements were rearranged to meet the demands of the pandemic while ensuring continued focus on learner progress [17]. In Canada, standardized guidance for medical schools resulted in more uniform withdrawals of learners from the clinical space [18]. Across Europe, guidelines directly shifted education online and in central and south America many educational experiences were suspended with a varied level of transition to online learning [19].Medical students in African countries may have suffered from a lack of similar templates to follow as there were few national or continent-wide policies and guidelines in place for reopening and education during shutdowns. Correspondingly, the responses across the African continent were more uneven, with many schools remaining closed for the duration of our survey period and with many schools reopening. This is in contrast to the US, where most medical schools followed guidance on timing from the Association of American Medical Schools [15]. Between March and July 2020, temporary mandates to close all universities (including medical schools) were issued in 44 of 54 countries in Africa [20, 21]. Our results add to this context, indicating that majority of medical students had their medical schools closed, with many students not able to resume their medical education even after several months at the end of our study period. Delays and cancellations of medical training have already caused some delay in the entrance of physicians into the workforce and this problem is likely to continue without intervention [21].

The pandemic related changes to medical education have likewise been detrimental to the environment for students. These findings confirm the myriad challenges facing teaching faculty at medical training institutions in Africa from COVID-19 and represent a significant opportunity to improve the learning environment. Most medical schools receive governmental grants to fund research and salary for their medical faculty and the projected economic contraction on the African continent is likely to cause limitations in funding for faculty members, further exacerbating the challenges in the learning environment [21]. Educators across the globe have suffered many new challenges because of the pandemic. In Indonesia, teachers working from home cite new difficulties covering the cost of electricity and internet access and report difficulties maintaining motivation [22]. In the United Kingdom, educators suffered from an increase in uncertainty and disruptions to their identity as teachers [23]. A study of educators in India found that in addition to technical support, teachers as well as students benefit from high-quality and timely interactions with each other [24]. Further support for both teachers and students during times of disruption appears to be needed to limit the harm from major societal stressors. For greatest efficacy, interventions should include financial and logistical support for educators as well as clear and collaborative communication and planning with teachers.

Students across Africa endorsed a shift to online learning and exams, although this transition appears to be less drastic than in HIC [25]. The large increase in online testing speaks to the need for robust and reliable internet access and online programming in Africa. Even before COVID-19, there had been a shift towards technology-integrated learning and self-directed work in many medical schools across the world. The pandemic has accelerated these trends, with varying levels of success [26]. However, many countries within Africa lack the reliable broadband access, consistent electricity and advanced smartphones necessary to access online learning content [27, 28]. A review of the literature suggests that many similar problems have been encountered worldwide. In South Asia, it is estimated that the shift to online learning was accelerated by 5-10 years [29]. There is a need to accommodate this accelerated transition to ensure adequate training of the healthcare workforce, where barriers to online learning have the potential to derail this trend. There are several initiatives currently underway to increase internet access across the continent with recommendations including: Removing roadblocks to infrastructure investment, promoting private sector investment, and involving high levels of political leadership [30, 31]. For the success and stability of future medical education in Africa, this access appears to be critical.

It is important to note that access to the internet is closely connected with many demographic and sociological factors. Notably, persons with disabilities are less likely to have access to computers or to the internet, after controlling for other sociodemographic factors [32]. Personal wealth also plays a large role in access to the internet, although some nations have been more successful at reducing this divide [33]. The digital divide is apparent both on a country level and between induvial within a nation [34–36]. It will be important to both improve country-level infrastructure capabilities and to provide targeted additional support to those sectors of the population who may face increased difficulties with internet connectivity.

The direct impact on student wellbeing in Africa is consistent with a recent global survey of young people that found a majority believe that the pandemic, school closures, and restricted social connections worsened pre-existing mental health conditions [37].

Our study had several important limitations. We distributed our survey though student networks, and our response rate varied by country and school, with some medical schools providing few respondents. This may be partially due to the variations in medical school prevalence, with 24 countries in sub-Saharan Africa having only one medical school, and 11 countries having no medical schools [4]. The average number of medical schools per country across Africa is only 1.8 schools [4]. While the use of social media allowed for a greater breadth of country and school respondents, this approach may have created a biased sample as those without access to social media during lockdowns and disruption would have been unable to participate. The prevalence of social media use across medical students in Africa is not known and would benefit from further study. The use of social media also did not allow calculation of the specific population reached by the survey instrument. Our data represent a snapshot of the experience of medical students in Africa to the unprecedented global stressor of COVID-19 and speaks to the need for a deeper assessment of individual-country responses. Additionally, qualitative research was not performed among medical students in Africa in order to inform survey creation and psychometric testing was not done to determine the underlying constructs of the instrument. Nonetheless, we believe these limitations are balanced by the need to rapidly develop and disseminate a highly relevant survey instrument to enable meaningful research that may benefit medical education and, therefore, public health during the COVID-19 pandemic.

Our study findings suggest a brewing problem in medical education across the African continent with an urgent need for solutions. Broadly, the outcomes mentioned here speak to the need for a comprehensive response plan in the event of future disruptions–COVID-19-related or otherwise–that encompasses, at minimum, mental health and logistical support for students and faculty, expanded internet access, and safety planning to minimize disruptions to student education.

## Conclusions

The response of medical schools to the COVID-19 pandemic has the potential to represent a transformational shift in education, a shift toward increased flexibility and adaptability, internet-based learning, and curricular innovation. However, this study highlights the urgent need for flexible and innovative approaches to continuing medical education in Africa. Without an effective mitigation strategy, the current disruption in medical training has the potential to impact medical care and the availability of healthcare workers across the continent in the years to come.

## Supplementary Information

**Additional file 1.**

## Data Availability

The datasets used and analysed during the current study are available from the corresponding author on reasonable request.
