# Supplementary material for: COVID-19 and medical education in Africa: a cross sectional analysis of the impact on medical students
Source: BMC Med Educ. 2021 Dec 9;21:605. doi: 10.1186/s12909-021-03038-3 (PMC8654489; doi:10.1186/s12909-021-03038-3)
Supplement: Supplementary file 1 — Additional file 1. [file 12909_2021_3038_MOESM1_ESM.docx]

Additional file 1: Full text of survey questions administered to 33 countries across Africa.
